# Validation of a brief screener for broad-spectrum mental and substance-use disorders in South Africa – ERRATUM

**DOI:** 10.1017/gmh.2024.109

**Published:** 2024-11-21

**Authors:** Melissa Ann Stockton, Ernesha Webb Mazinyo, Lungelwa Mlanjeni, Kwanda Nogemane, Nondumiso Ngcelwane, Annika C. Sweetland, Cale Neil Basaraba, Charl Bezuidenhout, Griffin Sansbury, Kathryn L. Lovero, David Olivier, Christoffel Grobler, Melanie M. Wall, Andrew Medina-Marino, Phumza Nobatyi, Milton L. Wainberg

**Keywords:** developing countries, assessment tools, psychometric evaluations, primary care, crosscultural

The publisher apologises that upon publication table 1 of Stockton MA, et al. 2024 was misnumbered, repeating 5 and 6 twice in Step Two Questions.

The correct table should have been as below:

**Table 1.** MwTool-13 Questions Definitions of a Positive Screen for Each Disorder Category

Numbering, Questions and Administration Instructions		Definition of a Positive Screen	Disorder
Step One Questions	1. In the last 2 weeks, how often have you been feeling down, depressed, or hopeless?	≥ “Several days” to any of the three questions	CMD
	2. In the last 2 weeks, how often have you been feeling nervous, anxious, or on edge?		Does not inform a specific disorder^a^
	3. In the last 2 weeks, how often have you been so restless that it’s hard to sit still?		CMD
POSITIVE to questions 1 or 2 or 3, CONTINUE SCREENING. If NEGATIVE for all three, STOP. If POSITIVE to question 2, but NEGATIVE to questions 5–11, refer to self–help.			
Step Two Questions	4. In the past year, how often do you have a drink containing alcohol?	Anyone (regardless of gender): ≥ “Between 2 and 4 times a month” on Q4 Women: “Monthly or less” on Q4 and ≥ “3 or 4” on Q5 Men: “Monthly or less” on Q4 and ≥ “5 or 6” on Q5	AUD
	5. In the past year, how many drinks containing alcohol do you have on a typical day when you are drinking?		
	6. In the past year, how many times have you used a recreational or illegal drug or used a prescription medication for non–medical reasons?	≥ “Once or twice”	SUD
	7. In the past year, have you ever felt that your thoughts were being directly interfered with or controlled by some outside force or person in a way that many people would find hard to believe (for instance, through telepathy)?	“Yes” to any of the four questions	SMD
	8. In the past year, have there been times when you felt that a group of people was plotting to cause you serious harm or injury?		
	9. In the past year, have there been times when you felt that something so strange was going on that other people would find it very hard to believe?		
	10. In the past year, did you at any time hear voices saying quite a few words or sentences when there was no one around that might account for it?		
	11. In the past month, have you wished you were dead or wished you could go to sleep and not wake up?	“Yes” to any of the three questions	SR
	12. In the past month, have you had any actual thoughts of killing yourself?		
	13. In the past 3 months, have you ever done anything, started to do anything, or prepared to do anything to end your life?		

Abbreviations: AUD, alcohol-use disorder; CMD, common mental disorder; SMD, severe mental disorder; SR, suicide risk; SUD, substance use disorder.

a While Q1-3 direct continuation to the step two questions for identifying AUD, SUD, and SMD, positive responses to Q1 and/or Q3 are considered indicative of CMD. Of note, endorsing only Q2 and none of the other questions is not indicative of a specific disorder.
