# Supplementary material for: Validation of a brief screener for broad-spectrum mental and substance-use disorders in South Africa
Source: Glob Ment Health (Camb). 2023 Dec 21;11:e4. doi: 10.1017/gmh.2023.89 (PMC10808975; doi:10.1017/gmh.2023.89)
Supplement: Stockton et al. supplementary material [file S2054425123000894sup001.docx]

**Annex 1: Description of the MINI-V adaptation**

Most of the MINI modules administered came directly from the MINI-V. However, for some disorders we made small modifications or use the MINI-Plus module for brevity. For each module administered, we describe the source (MINI-V or MINI-Plus) and any modifications made below.

- Major Depressive Episode, MINI-V
- Dysthymia, MINI-V
- Suicidality, MINI-Plus
- (Hypo) Manic Episode, MINI-V, excluding questions about past episode
- Panic Disorder, MINI-V
- Obsessive-Compulsive Disorder, MINI-V
- PTSD, MINI-Plus, excluding the question “Did you respond with intense fear, helplessness or horror.”
- Alcohol Abuse or Dependence, MINI-V
- Non-Alcohol Psychoactive Substance Use Disorders, MINI-V, with a revised list of drugs to appropriately categorize and include local drugs
- Psychotic Disorders, MINI-V, excluding questions relevant to mood disorder with psychotic features lifetime & current
- Generalized Anxiety Disorder, MINI-V excluding the administrator prompt “Is the patient’s anxiety restricted exclusively to, or better explained by, any disorder prior to this point?”

# Annex 2: Mental Wellness Tool-13 (mwTool-13) Questionnaire


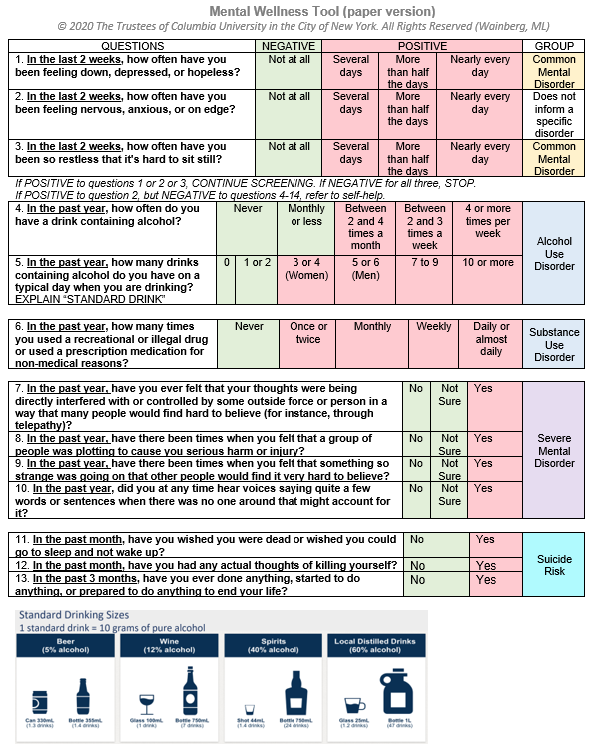


# Annex 3: MwTool-13 restricted and stratified analyses using the original two-step administration and definitions of a positive screen

Note: These are companion tables to table 5.

Table 1: Performance of the mwTool-13 using the two-step administration method and the original definitions of a positive screen for each disorder category among the Total population and restrict to those who received the tool in isiXhosa

| Disorder | Total Population (n=1885) | | isiXhosa (n=1,792) | |
| --- | --- | --- | --- | --- |
|  | Sensitivity (95%CI) | Specificity (95%CI) | Sensitivity (95%CI) | Specificity (95%CI) |
| *Step Two Questions* |  |  |  |  |
| Any Disorder | 83.36 (80.32-86.1) | 46.45 (43.61-49.31) | 83.1 (79.96-85.92) | 46.31 (43.4-49.24) |
| CMD | 91.48 (88.54-93.87) | 44.57 (41.97-47.19) | 91.84 (88.84-94.25) | 44.53 (41.87-47.22) |
| CMD | 88.86 (85.62-91.6) | 47.72 (45.1-50.35) | 89.28 (85.96-92.04) | 47.69 (45.01-50.38) |
| Major Depressive Episode | 90.89 (87.66-93.5) | 46.99 (44.42-49.57) | 91.34 (88.05-93.96) | 46.99 (44.36-49.63) |
| Generalized Anxiety Disorder | 90.08 (83.32-94.77) | 40.82 (38.51-43.15) | 90.68 (83.93-95.25) | 40.92 (38.55-43.32) |
| PTSD | 89.57 (83.83-93.81) | 41.52 (39.18-43.89) | 90.38 (84.64-94.52) | 41.63 (39.22-44.06) |
| *Step One Questions* |  |  |  |  |
| AUD | 56.67 (49.09-64.02) | 84.50 (82.69-86.19) | 55.23 (47.48-62.81) | 84.43 (82.57-86.16) |
| SUD | 64.52 (45.37-80.77) | 93.42 (92.19-94.51) | 64.52 (45.37-80.77) | 93.98 (92.77-95.05) |
| SMD | 71.71 (63.84-78.71) | 61.92 (59.58-64.21) | 71.92 (63.89-79.03) | 61.6 (59.2-63.96) |
| Psychotic Disorder | 67.37 (56.98-76.64) | 60.61 (58.31-62.89) | 67.03 (56.39-76.53) | 60.26 (57.89-62.59) |
| Manic or hypomanic episode | 77.61 (65.78-86.89) | 60.56 (58.27-62.82) | 78.46 (66.51-87.69) | 60.28 (57.93-62.6) |
| SR | 86.84 (79.23-92.44) | 80.24 (78.3-82.07) | 85.85 (77.74-91.86) | 80.55 (78.57-82.41) |

Table 2: Performance of the mwTool-13 using the two-step administration method and the original definitions of a positive screen for each disorder category, by gender

| Disorder | Men (n=651) | | Women (n=1,232) | |
| --- | --- | --- | --- | --- |
|  | Sensitivity (95%CI) | Specificity (95%CI) | Sensitivity (95%CI) | Specificity (95%CI) |
| *Step One Questions* |  |  |  |  |
| Any Disorder | 72.80 (66.69-78.34) | 53.64 (48.69-58.54) | 89.15 (85.83-91.91) | 42.80 (39.34-46.32) |
| CMD | 82.24 (73.67-88.96) | 49.08 (44.8-53.37) | 94.29 (91.31-96.47) | 41.84 (38.56-45.17) |
| CMD | 78.5 (69.51-85.86) | 52.21 (47.91-56.47) | 92 (88.65-94.62) | 45.01 (41.69-48.36) |
| Major Depressive Episode | 80.65 (71.15-88.11) | 51.79 (47.56-56.01) | 93.91 (90.65-96.29) | 44.13 (40.89-47.41) |
| Generalized Anxiety Disorder | 86.96 (66.41-97.22) | 48.41 (44.43-52.4) | 90.72 (83.12-95.67) | 36.65 (33.84-39.53) |
| PTSD | 83.33 (65.28-94.36) | 48.63 (44.63-52.64) | 90.91 (84.66-95.21) | 37.55 (34.67-40.48) |
| *Step Two Questions* |  |  |  |  |
| AUD | 41.33 (30.08-53.3) | 85.59 (82.45-88.36) | 67.62 (57.79-76.43) | 83.94 (81.66-86.04) |
| SUD | 69.23 (48.21-85.67) | 93.92 (91.75-95.66) | 40 (5.27-85.34) | 93.15 (91.59-94.5) |
| SMD | 67.95 (56.42-78.07) | 69.11 (65.15-72.87) | 75.68 (64.31-84.9) | 58.46 (55.56-61.32) |
| Psychotic Disorder | 71.21 (58.75-81.7) | 68.72 (64.79-72.46) | 58.62 (38.94-76.48) | 56.77 (53.92-59.6) |
| Manic or hypomanic episode | 60 (36.05-80.88) | 65.45 (61.6-69.16) | 85.11 (71.69-93.8) | 58.06 (55.19-60.89) |
| SR | 80.00 (56.34-94.27) | 84.47 (81.40-87.21) | 88.30 (80.03-94.01) | 77.94 (75.42-80.32) |

*Excludes the two individuals who responded “Transgender” and “Non-binary”

Table 3: Performance of the mwTool-13 using the two-step administration method and the original definitions of a positive screen for each disorder category, by self-reported HIV status

| Disorder | HIV Positive (n=487) | | HIV Negative (n=1,195) | |
| --- | --- | --- | --- | --- |
|  | Sensitivity (95%CI) | Specificity (95%CI) | Sensitivity (95%CI) | Specificity (95%CI) |
| *Step One Questions* |  |  |  |  |
| Any Disorder | 85.22 (79.58-89.80) | 43.31 (37.47-49.29) | 83.63 (79.58-87.16) | 49.75 (46.24-53.27) |
| CMD | 94.81 (90.02-97.73) | 43.54 (38.15-49.06) | 91.57 (87.4-94.7) | 46.83 (43.61-50.07) |
| CMD | 91.56 (86-95.43) | 46.85 (41.39-52.36) | 89.56 (85.08-93.06) | 49.89 (46.66-53.13) |
| Major Depressive Episode | 93.53 (88.06-97) | 45.98 (40.65-51.37) | 91.32 (86.78-94.7) | 49.08 (45.9-52.26) |
| Generalized Anxiety Disorder | 95.35 (84.19-99.43) | 37.61 (33.09-42.3) | 89.55 (79.65-95.7) | 43.53 (40.61-46.48) |
| PTSD | 89.66 (78.83-96.11) | 38 (33.38-42.77) | 90.59 (82.29-95.85) | 44.14 (41.2-47.12) |
| *Step Two Questions* |  |  |  |  |
| AUD | 60.00 (47.59-71.53) | 81.45 (77.37-85.07) | 52.17 (41.50-62.70) | 85.31 (83.08-87.35) |
| SUD | 57.14 (18.41-90.1) | 93.75 (91.2-95.74) | 73.68 (48.8-90.85) | 94.13 (92.63-95.41) |
| SMD | 70.00 (50.60-85.27) | 53.83 (49.14-58.47) | 72.82 (63.16-81.12) | 64.01 (61.08-66.86) |
| Psychotic Disorder | 54.55 (23.38-83.25) | 52.52 (47.93-57.08) | 68.57 (56.37-79.15) | 62.67 (59.77-65.50) |
| Manic or hypomanic episode | 76.19 (52.83-91.78) | 53.65 (49-58.25) | 80.00 (64.35-90.95) | 62.25 (59.38-65.06) |
| SR | 89.13 (76.43-96.38) | 79.82 (75.76-83.47) | 84.75 (73.01-92.78) | 81.51 (79.13-83.73) |

*Excludes those who “didn’t know” and “refused to answer.”

Table 4: Performance of the mwTool-13 using the two-step administration method and the original definitions of a positive screen for each disorder category, by self-reported lifetime TB history

| Disorder | Ever had TB (n=284) | | Never had TB (n=1,601) | |
| --- | --- | --- | --- | --- |
|  | Sensitivity (95%CI) | Specificity (95%CI) | Sensitivity (95%CI) | Specificity (95%CI) |
| *Step One Questions* |  |  |  |  |
| Any Disorder | 75.00 (65.93-82.70) | 52.91 (45.16-60.55) | 85.03 (81.80-87.88) | 45.38 (42.33-48.47) |
| CMD | 89.04 (79.54-95.15) | 52.61 (45.64-59.5) | 91.95 (88.77-94.46) | 43.17 (40.37-46.01) |
| CMD | 84.93 (74.64-92.23) | 55.92 (48.95-62.73) | 89.61 (86.12-92.47) | 46.30 (43.47-49.15) |
| Major Depressive Episode | 85.29 (74.61-92.72) | 55.09 (48.2-61.85) | 92.01 (88.59-94.67) | 45.61 (42.83-48.40) |
| Generalized Anxiety Disorder | 92.31 (63.97-99.81) | 47.23 (41.16-53.36) | 89.81 (82.51-94.8) | 39.65 (37.16-42.18) |
| PTSD | 94.12 (71.31-99.85) | 47.94 (41.81-54.11) | 89.04 (82.81-93.6) | 40.34 (37.81-42.92) |
| *Step Two Questions* |  |  |  |  |
| AUD | 45.65 (30.9-60.99) | 85.71 (80.61-89.9) | 60.45 (51.64-68.78) | 84.30 (82.33-86.13) |
| SUD | 63.64 (30.79-89.07) | 93.77 (90.22-96.33) | 65 (40.78-84.61) | 93.36 (92.02-94.54) |
| SMD | 78.57 (49.2-95.34) | 64.44 (58.42-70.15) | 71.01 (62.69-78.42) | 61.45 (58.9-63.95) |
| Psychotic Disorder | 71.43 (29.04-96.33) | 63.18 (57.2-68.87) | 67.05 (56.21-76.7) | 60.15 (57.63-62.62) |
| Manic or hypomanic episode | 87.5 (47.35-99.68) | 63.77 (57.79-69.44) | 76.27 (63.41-86.38) | 59.99 (57.49-62.44) |
| SR | 94.12 (71.31-99.85) | 82.4 (77.29-86.77) | 85.57 (76.97-91.88) | 79.85 (77.74-81.85) |

*Ever had TB includes those who responded “Currently,” “Yes, in the last two years,” and Yes, more than two years ago.”

# Annex 4: Considerations for modifying the MwTool-13 to improve the performance of the AUD and SUD questions

Step 1: Assess changing the definition of a positive screen for AUD

To examine changing the definition of a positive screen, we considered the AUD questions’ performance using less-stringent cut-offs for frequency (Q4, “at least monthly or less” or “2-4 times a month,” regardless of gender) and/or amount (Q5, at least “1-2 drinks” or “3-4 drinks”, regardless of gender) for both questions together and individually. (**Annex 4, Table 1**) Ultimately, these changes failed to adequately improve the sensitivity, which remained less than 70%.

| Annex 4, Table 1: Comparing the performance of the AUD questions using different definitions of a positive screen when included in the step two questions | | |
| --- | --- | --- |
| AUD Questions  (Definition of Positive Screen) | Sensitivity (95%CI) | Specificity (95%CI) |
| Q4 & Q5 (Frequency and Amount, Original^α^) | 56.67 (49.09-64.02) | 84.5 (82.69-86.19) |
| Q4 – (Frequency, ≥ Monthly or Less) or Q5 (Amount, ≥1 or 2) | 66.11 (58.7-72.99) | 76.01 (73.91-78.02) |
| Q4 – (Frequency, ≥ Monthly or Less) | 66.11 (58.7-72.99) | 76.48 (74.39-78.48) |
| Q4 – (Frequency, ≥ 2-4 times a month) | 30 (23.41-37.26) | 93.61 (92.34-94.72) |
| Q5 – (Amount, ≥1 or 2, everyone) | 66.11 (58.7-72.99) | 76.6 (74.51-78.59) |
| Q5 – (Amount, ≥3 or 4, everyone) | 52.78 (45.21-60.25) | 83.75 (81.92-85.47) |
| Q5 – (Amount, ≥3 or 4, women; ≥5 or 6, men ^α^) | 50 (42.47-57.53) | 86.04 (84.3-87.65) |
| AUD=Alcohol Use Disorder  Q4 – Drinking Frequency: In the past year, how often do you have a drink containing alcohol?  Q5 – Drinking Amount: In the past year, how many drinks containing alcohol do you have on a typical day when you are drinking?  αThose who did not self-identify as male or female are treated as missing (n=2).  The original definition of a positive screen:   - anyone (regardless of gender) who reported drinking more often than “monthly or less” over the past year on Q4 (Drinking Frequency); - women who reported both drinking “monthly or less” over the past year on Q4 (Drinking Frequency) and drinking 3 or more drinks on a typical drinking day on Q5 (Drinking Amount) - men who reported both drinking at least “monthly or less” over the past year on Q4 (Drinking Frequency) and reported drinking 5 or more drinks on a typical drinking day on Q5 (Drinking Amount). | | |

Step 2: Assess changing the administration by including the AUD questions in the step one question set

We then examined whether adding the AUD questions to the step one question set would improve performance. When added to the initial question set, using the original definitions for a positive screen yielded a sensitivity of 76.67% (95% CIs: 73.77-85.67) for AUD **(Annex 4, Table 2)**, significantly higher than in the original two-step administration which yielded 56.67 (95% CIs: 49.09-64.02). **(Annex 4, Table 1)** However, using a single item (either Q4 or Q5 for drinking frequency or amount) added to the initial question set with less stringent definition of a positive screen yielded similar or improved sensitivity. Ultimately, using only Q5 (Drinking Amount) with a cut-off of ≥3 or 4 regardless of gender yielded comparably high sensitivity [74.44% (95% CIs: 67.42-80.64)] to the original definition [76.67% (95% CIs: 69.8-82.64)], enhancing face-validity without compromising sensitivity.

| Annex 4, Table 2: Comparing the performance of the AUD questions using different definitions of a positive screen when included in the step one question set | | |
| --- | --- | --- |
| AUD Questions  (Definition of Positive Screen) | Sensitivity (95%CI) | Specificity (95%CI) |
| Q4 & Q5 (Frequency and Amount, Original^α^) | 76.67 (69.80-82.64) | 76.16 (74.06-78.17) |
| Q4 – (Frequency, ≥ Monthly or Less) or Q5 (Amount, ≥1 or 2) | 94.44 (90.02-97.3) | 62.17 (59.82-64.48) |
| Q4 – (Frequency, ≥ Monthly or Less) | 92.22 (87.29-95.68) | 63.17 (60.83-65.46) |
| Q4 – (Frequency, ≥ 2-4 times a month) | 45.56 (38.13-53.13) | 88.62 (87.02-90.09) |
| Q5 – (Amount, ≥1 or 2, everyone) | 94.44 (90.02-97.30) | 62.87 (60.53-65.17) |
| Q5 – (Amount, ≥3 or 4, everyone) | 74.44 (67.42-80.64) | 75.54 (73.43-77.57) |
| Q5 – (Amount, ≥3 or 4, women; ≥5 or 6, men ^α^) | 68.33 (61.00-75.05) | 78.94 (76.93-80.86) |
| AUD=Alcohol Use Disorder  Q4 – Drinking Frequency: In the past year, how often do you have a drink containing alcohol?  Q5 – Drinking Amount: In the past year, how many drinks containing alcohol do you have on a typical day when you are drinking?  αThose who did not self-identify as male or female are treated as missing (n=2).  The original definition of a positive screen:   - anyone (regardless of gender) who reported drinking more often than “monthly or less” over the past year on Q4 (Drinking Frequency); - women who reported both drinking “monthly or less” over the past year on Q4 (Drinking Frequency) and drinking 3 or more drinks on a typical drinking day on Q5 (Drinking Amount)   men who reported both drinking at least “monthly or less” over the past year on Q4 (Drinking Frequency) and reported drinking 5 or more drinks on a typical drinking day on Q5 (Drinking Amount). | | |

Step 3: Assess changing the administration by including the SUD question in the step one question set

Given the original definition of a positive screen for SUD considered any endorsement of the SUD question (Q6) as indicative of SUD, we could only improve the question’s performance by including it in the step one question set. When included in the initial question set, the SUD question yielded a sensitivity of 80.65% (95% CIs: 62.53-92.550), a substantial improvement over 64.52% (95% CIs: 45.37-80.77) when included in the step two questions. Given the small prevalence of SUD (1.6%) and the potential decrease in brevity to the first step, we ultimately decided it would be imprudent to change the administration of SUD question based on the available data.

Step 4: Assess the impact of including a single AUD question (Q5) in the step one question set on the performance of the questions for identifying any disorder, SUD, SMD, and SR

To promote the brevity of the first step and simplicity in administration, we assessed how including the drinking amount question (Q5) in the step one question set would impact the performance of the step two questions. Including Q5 in the initial question set modestly improved the sensitivity of the tool identifying any disorder, SMD, SUD, and SR, yielding sensitivities >70%. (**Annex 4, Table 3**). Of note, while an endorsement of the original step one question set (Q1-Q3) yielded similar sensitivity to the modified step one question set (Q1-Q3, Q5), the original step one question set yielded higher specificity [46.56% (95%CI: 43.72-49.42)] than the modified [38.08% (95%CI: 35.33-40.89)].

| Annex 4, Table 3: Comparing the performance of the original and modified mwTool in identifying any disorder, SUD, SMD, and SR | | | | |
| --- | --- | --- | --- | --- |
| Disorder | Questions | Mini Prev. | Sensitivity  (95% CIs) | Specificity  (95% CIs) |
| **Original mwTool-13** |  |  |  |  |
| *Step One Questions* |  |  |  |  |
| Any Disorder | Q1-Q3 | 36% | 83.36 (80.32-86.10) | 46.45 (43.61-49.31) |
| *Step Two Questions* |  |  |  |  |
| SUD | Q6 | 2% | 64.52 (45.37-80.77) | 93.42 (92.19-94.51) |
| SMD | Q7-Q10 | 8% | 71.71 (63.84-78.71) | 61.92 (59.58-64.21) |
| SR | Q11-Q13 | 6% | 86.84 (79.23-92.44) | 80.24 (78.3-82.07) |
| **SA-mwTool-12** | | | | |
| *Modified Step One Questions* | | | | |
| Any Disorder | Q1-Q3, Q5* | 36% | 89.75 (87.20-91.93) | 38.03 (35.29-40.83) |
| *Modified Step Two Questions* |  |  |  |  |
| SUD | Q6 | 2% | 74.19 (55.39-88.14) | 91.69 (90.34-92.91) |
| SMD | Q7-Q10 | 8% | 75.94 (69.16-81.87) | 62.01 (59.66-64.33) |
| SR | Q11-Q13 | 6% | 88.60 (81.29-93.79) | 79.39 (77.43-81.25) |
| Any disorder includes CMD, AUD, SUD, SMD, and/or SR; SUD=Substance Use Disorder; SMD=Severe Mental Disorder; SR=Suicide Risk *Positive screen is defined as endorsing any of Q1-Q3 or responding ≥3 or 4 to Q5 | | | | |

# Annex 3: SA-mwTool-12 restricted and stratified analyses using the original two-step administration and definitions of a positive screen

Note: These are companion tables to table 6.

Annex 3, Table 1: Performance of the SA-mwTool-12 using the two-step administration method and the original definitions of a positive screen for each disorder category among the Total population and restrict to those who received the tool in isiXhosa

| Disorder | Total Population (n=1885) | | isiXhosa (n=1,792) | |
| --- | --- | --- | --- | --- |
|  | Sensitivity (95%CI) | Specificity (95%CI) | Sensitivity (95%CI) | Specificity (95%CI) |
| *Step One Questions* |  |  |  |  |
| Any Disorder | 89.75 (87.20-91.93) | 38.03 (35.29-40.83) | 89.67 (87.04-91.92) | 37.90 (35.09-40.77) |
| CMD | 91.48 (88.54-93.87) | 44.57 (41.97-47.19) | 91.84 (88.84-94.25) | 44.53 (41.87-47.22) |
| CMD | 88.86 (85.62-91.6) | 47.72 (45.1-50.35) | 89.28 (85.96-92.04) | 47.69 (45.01-50.38) |
| Major Depressive Episode | 90.89 (87.66-93.5) | 46.99 (44.42-49.57) | 91.34 (88.05-93.96) | 46.99 (44.36-49.63) |
| Generalized Anxiety Disorder | 90.08 (83.32-94.77) | 40.82 (38.51-43.15) | 90.68 (83.93-95.25) | 40.92 (38.55-43.32) |
| PTSD | 89.57 (83.83-93.81) | 41.52 (39.18-43.89) | 90.38 (84.64-94.52) | 41.63 (39.22-44.06) |
| AUD | 74.44 (67.42-80.64) | 75.54 (73.43-77.57) | 73.84 (66.6-80.23) | 75.43 (73.26-77.51) |
| *Step Two Questio* |  |  |  |  |
| SUD | 74.19 (55.39-88.14) | 91.69 (90.34-92.91) | 74.19 (55.39-88.14) | 92.39 (91.05-93.59) |
| SMD | 75.94 (69.16-81.87) | 62.01 (59.66-64.33) | 73.33 (65.34-80.26) | 60.75 (58.35-63.12) |
| Psychotic Disorder | 68.42 (58.08-77.58) | 59.66 (57.35-61.95) | 68.13 (57.53-77.51) | 59.38 (57.00-61.72) |
| Manic or hypomanic episode | 79.10 (67.43-88.08) | 59.63 (57.32-61.89) | 80.00 (68.23-88.89) | 59.41 (57.05-61.73) |
| SR | 88.6 (81.29-93.79) | 79.39 (77.43-81.25) | 87.74 (79.94-93.31) | 79.72 (77.72-81.61) |

Table 2: Performance of the SA-mwTool-12 using the two-step administration method and the original definitions of a positive screen for each disorder category, by gender

| Disorder | Men (n=651) | | Women (n=1,232) | |
| --- | --- | --- | --- | --- |
|  | Sensitivity (95%CI) | Specificity (95%CI) | Sensitivity (95%CI) | Specificity (95%CI) |
| *Step One Questions* |  |  |  |  |
| Any Disorder | 86.19 (81.16-90.30) | 39.81 (35.05-44.71) | 91.68 (88.67-94.11) | 37.17 (33.81-40.63) |
| CMD | 82.24 (73.67-88.96) | 49.08 (44.8-53.37) | 94.29 (91.31-96.47) | 41.84 (38.56-45.17) |
| CMD | 78.5 (69.51-85.86) | 52.21 (47.91-56.47) | 92 (88.65-94.62) | 45.01 (41.69-48.36) |
| Major Depressive Episode | 80.65 (71.15-88.11) | 51.79 (47.56-56.01) | 93.91 (90.65-96.29) | 44.13 (40.89-47.41) |
| Generalized Anxiety Disorder | 86.96 (66.41-97.22) | 48.41 (44.43-52.4) | 90.72 (83.12-95.67) | 36.65 (33.84-39.53) |
| PTSD | 83.33 (65.28-94.36) | 48.63 (44.63-52.64) | 90.91 (84.66-95.21) | 37.55 (34.67-40.48) |
| AUD | 76 (64.75-85.11) | 100 (99.36-100) | 73.33 (63.81-81.49) | 78.70 (76.20-81.06) |
| *Ste Two Questions* |  |  |  |  |
| SUD | 80.77 (60.65-93.45) | 91.52 (89.05-93.58) | 40.00 (5.27-85.34) | 91.77 (90.09-93.25) |
| SMD | 69.23 (57.76-79.19) | 68.59 (64.61-72.37) | 77.03 (65.79-86.01) | 57.34 (54.43-60.21) |
| Psychotic Disorder | 72.73 (60.36-82.97) | 68.21 (64.26-71.96) | 58.62 (38.94-76.48) | 55.61 (52.75-58.44) |
| Manic or hypomanic episode | 60.00 (36.05-80.88) | 64.82 (60.95-68.55) | 87.23 (674.25-95.17) | 56.96 (54.08-59.80) |
| SR | 85 (62.11-96.79) | 83.20 (80.05-86.04) | 89.36 (81.3-94.78) | 77.33 (74.78-79.73) |

*Excludes the two individuals who responded “Transgender” and “Non-binary”

Table 3: Performance of the SA-mwTool-12 using the two-step administration method and the original definitions of a positive screen for each disorder category, by self-reported HIV status

| Disorder | HIV Positive (n=487) | | HIV Negative (n=1,195) | |
| --- | --- | --- | --- | --- |
|  | Sensitivity (95%CI) | Specificity (95%CI) | Sensitivity (95%CI) | Specificity (95%CI) |
| *Step One Questions* |  |  |  |  |
| Any Disorder | 90.64 (85.77-94.27) | 31.34 (25.99-37.08) | 90.54 (87.20-93.25) | 41.92 (38.48-45.41) |
| CMD | 94.81 (90.02-97.73) | 43.54 (38.15-49.06) | 91.57 (87.40-94.70) | 46.83 (43.61-50.07) |
| CMD | 91.56 (86-95.43) | 46.85 (41.39-52.36) | 89.56 (85.08-93.06) | 49.89 (46.66-53.13) |
| Major Depressive Episode | 93.53 (88.06-97) | 45.98 (40.65-51.37) | 91.32 (86.78-94.7) | 49.08 (45.9-52.26) |
| Generalized Anxiety Disorder | 95.35 (84.19-99.43) | 37.61 (33.09-42.3) | 89.55 (79.65-95.7) | 43.53 (40.61-46.48) |
| PTSD | 89.66 (78.83-96.11) | 38 (33.38-42.77) | 90.59 (82.29-95.85) | 44.14 (41.2-47.12) |
| AUD | 74.29 (62.44-83.99) | 71.22 (66.62-75.52) | 71.74 (61.39-80.64) | 76.79 (74.18-79.25) |
| *Step Two Questions* |  |  |  |  |
| SUD | 57.14 (18.41-90.1) | 91.67 (88.83-93.98) | 84.21 (60.42-96.62) | 92.52 (90.86-93.96) |
| SMD | 76.67 (57.71-90.01) | 52.95 (48.26-57.61) | 72.82 (63.16-81.12) | 72.81 (63.16-81.12) |
| Psychotic Disorder | 63.64 (30.79-89.07) | 51.47 (46.88-56.04) | 68.57 (56.37-79.15) | 61.69 (58.78-64.54) |
| Manic or hypomanic episode | 80.95 (58.09-94.55) | 52.57 (47.93-55.19) | 80.00 (6.32-90.94) | 61.30 (58.42-64.12) |
| SR | 89.13 (76.43-96.38) | 79.37 (75.28-83.05) | 88.14 (77.07-95.09) | 80.55 (78.12-82.81) |

*Excludes those who “didn’t know” and “refused to answer.”

Table 4: Performance of the SA-mwTool-12 using the two-step administration method and the original definitions of a positive screen for each disorder category, by self-reported lifetime TB history

| Disorder | Ever had TB (n=284) | | Never had TB (n=1,601) | |
| --- | --- | --- | --- | --- |
|  | Sensitivity (95%CI) | Specificity (95%CI) | Sensitivity (95%CI) | Specificity (95%CI) |
| *Step One Questions* |  |  |  |  |
| Any Disorder | 88.39 (80.97-93.67) | 30.23 (23.47-37.69) | 90.01 (87.23-92.37) | 39.32 (36.34-42.37) |
| CMD | 89.04 (79.54-95.15) | 52.61 (45.64-59.5) | 91.95 (88.77-94.46) | 43.17 (40.37-46.01) |
| CMD | 84.93 (74.64-92.23) | 55.92 (48.95-62.73) | 89.61 (86.12-92.47) | 46.30 (43.47-49.15) |
| Major Depressive Episode | 85.29 (74.61-92.72) | 55.09 (48.2-61.85) | 92.01 (88.59-94.67) | 45.61 (42.83-48.4) |
| Generalized Anxiety Disorder | 92.31 (63.97-99.81) | 47.23 (41.16-53.36) | 89.81 (82.51-94.8) | 39.65 (37.16-42.18) |
| PTSD | 94.12 (71.31-99.85) | 47.94 (41.81-54.11) | 89.04 (82.81-93.6) | 40.34 (37.81-42.92) |
| AUD | 78.26 (63.64-89.05) | 66.39 (60-72.36) | 73.88 (65.59-81.08) | 77.03 (74.79-79.16) |
| *Step Two Questions* |  |  |  |  |
| SUD | 72.73 (39.03-93.98) | 90.11 (85.94-93.38) | 75.00 (50.9-91.34) | 91.97 (90.52-93.26) |
| SMD | 85.71 (57.19-98.22) | 63.33 (57.28-69.09) | 71.74 (63.45-79.07) | 56.69 (54.19-59.16) |
| Psychotic Disorder | 85.71 (42.13-99.64) | 62.09 (56.10-67.83) | 67.05 (56.21-76.7) | 59.22 (56.69-61.71) |
| Manic or hypomanic episode | 87.50 (47.35-99.68) | 62.32 (56.31-68.06) | 77.97 (65.27-87.71) | 59.14 (56.64-61.61) |
| SR | 94.12 (71.31-99.85) | 80.15 (74.85-84.76) | 87.63 (79.39-93.44) | 79.26 (77.12-81.28) |

*Ever had TB includes those who responded “Currently,” “Yes, in the last two years,” and Yes, more than two years ago.”

**Annex 6: Modified South African Mental Wellness Tool: SA-mwTool-12**

| Updated Numbering, Questions and Administration Instructions | | Definition of a Positive Screen | Disorder |
| --- | --- | --- | --- |
| Step One Questions | 1. In the last 2 weeks, how often have you been feeling down, depressed, or hopeless? | Reporting “several days” or more to Q1, Q2, or Q3 | CMD |
|  | 2. In the last 2 weeks, how often have you been feeling nervous, anxious, or on edge? |  | Does not inform a specific disorder* |
|  | 3. In the last 2 weeks, how often have you been so restless that it's hard to sit still? |  | CMD |
|  | 4. In the past year, how many drinks containing alcohol do you have on a typical day when you are drinking? | Reporting “3 or 4,” regardless of gender | AUD |
| POSITIVE to questions 1, 2, 3, and/or 4 CONTINUE SCREENING.  If NEGATIVE for all four, STOP.  If POSITIVE to question 2, but NEGATIVE to questions 5-12, refer to self-help. | | | |
| Step Two Questions | 5. In the past year, how many times have you used a recreational or illegal drug or used a prescription medication for non-medical reasons? | ≥ “Once or twice” | SUD |
|  | 6. In the past year, have you ever felt that your thoughts were being directly interfered with or controlled by some outside force or person in a way that many people would find hard to believe (for instance, through telepathy)? | Reporting “yes” to any of the four questions | SMD |
|  | 7. In the past year, have there been times when you felt that a group of people was plotting to cause you serious harm or injury? |  |  |
|  | 8. In the past year, have there been times when you felt that something so strange was going on that other people would find it very hard to believe? |  |  |
|  | 9. In the past year, did you at any time hear voices saying quite a few words or sentences when there was no one around that might account for it? |  |  |
|  | 10. In the past month, have you wished you were dead or wished you could go to sleep and not wake up? | Reporting “yes” any of the three questions | SR |
|  | 11. In the past month, have you had any actual thoughts of killing yourself? |  |  |
|  | 12. In the past 3 months, have you ever done anything, started to do anything, or prepared to do anything to end your life? |  |  |

CMD=Common Mental Disorder; AUD=Alcohol Use Disorder, SUD=Substance Use Disorder; SMD=Severe Mental Disorder; SR=Suicide Risk; *While Q1-4 are used indicate continuing on to the step two questions, only Q1 and Q3 are used to identify CMD. Of note, endorsing only Q2 and none of the other questions is not indicative of a specific disorder.
